# A Review of the Value of Procalcitonin as a Marker of Infection

**DOI:** 10.7759/cureus.1148

**Published:** 2017-04-10

**Authors:** Richard Taylor, Adriana Jones, Steven Kelly, Michael Simpson, Jordan Mabey

**Affiliations:** 1 Medical Student, University of Central Florida College of Medicine

**Keywords:** procalcitonin, pro-inflammatory marker, systematic review, sepsis

## Abstract

Septicemia is a growing problem within the United States (US), which increases mortality and the cost of care. Procalcitonin is a pro-inflammatory marker that could be useful in the diagnosis of infection. In the past, procalcitonin levels have been evaluated to diagnose sepsis or guide antibiotic therapy, but it was not determined if it would differentiate between sepsis and other causes of inflammation. Studies reviewed here showed procalcitonin to be a useful biomarker as an indication of bacterial infection. Infections can be diagnosed earlier and managed appropriately to avoid progression to septicemia, reduce mortality, and overall medical costs.

## Introduction and background

Septicemia is a growing problem within the United States (US) healthcare system. There exists speculation as to the root cause of this increase, but a 2016 report by the Agency for Health Research and Quality (AHRQ) found that sepsis is the most expensive condition treated in the US and by a wide margin [1]. The data available from 2013 found that aggregate hospital costs for treating sepsis were over 23 billion dollars, while osteoarthritis, which was second, cost over 16 billion dollars. Additionally, a previous report using data from 2011 found that septicemia was second only to congestive heart failure in the number of all-cause 30-day hospital readmissions [2].

When compared to reports from previous years by the same government agency, the costs only seem to be climbing for treating sepsis in the US [3]. It is for this reason that novel ways to screen for and monitor sepsis are vital. Any decrease in the length of hospital stay or cost of treatment could have profound impacts on the overall treatment costs in this country. Here, we seek to understand the potential role that procalcitonin may have as a marker for sepsis that may be able to guide treatment of this condition.

Procalcitonin is the peptide precursor of the hormone calcitonin. It is well-known to be produced by the parafollicular cells of the thyroid, but it is also secreted from neuroendocrine cells of the lung and intestine. The latter two sources of procalcitonin provide its true clinical utility, as they increase its production in response to a proinflammatory stimulus, particularly when the stimulus is of bacterial origin. While the baseline levels in most adults are < 10 pg/mL, the levels of procalcitonin can increase to more than 400 times baseline (> 4,000 pg/mL) rapidly when an endotoxin enters the bloodstream [4]. This has led to the suggestion that procalcitonin may have a potentially useful clinical application to decrease mortality in those who present with signs of infection by monitoring for the onset and resolution of sepsis.

Previously, researchers have compared calcitonin with other inflammatory markers to determine which provides the greatest sensitivity and specificity for sepsis and can differentiate it from similar conditions, such as systemic inflammatory response syndrome. One 2007 study raised concerns regarding the specificity of procalcitonin as an indicator of sepsis. Researchers from the study concluded that “procalcitonin cannot reliably differentiate sepsis from other non-infectious causes of systemic inflammatory response syndrome in critically ill adult patients” [5]. Their findings, however, led to comparison studies with other markers of inflammation in later years, and in 2012, a different inflammatory marker, CD64, was found to be slightly more sensitive and specific than procalcitonin for identification of sepsis. However, these researchers commented on the utility of procalcitonin and, therefore, recommended that a combination panel of CD64, procalcitonin, and a third inflammatory marker, a soluble triggering receptor expressed on myeloid cells-1 (sTREM-1), should be used to most reliably differentiate sepsis from other causes of systemic inflammation [6].

Additionally, researchers in the past have evaluated procalcitonin levels to guide antibiotic therapy with the goal of decreasing the overall duration of antibiotic treatment in intensive care unit (ICU) patients. The conclusions drawn from these studies have been consistent in their findings but heterogenous in their recommendations. For instance, a 2011 meta-analysis concluded that “measurement of procalcitonin levels for antibiotic decisions in patients with respiratory tract infections and sepsis reduces antibiotic exposure without worsening the mortality rate” and suggested algorithms to follow to guide therapy [7]. However, a large randomized control trial in 2014 concluded that while using procalcitonin-based algorithms does reduce the length of antibiotic therapy, it may not do so to an appreciable extent and that all-cause mortality remains unchanged [8].

## Review

The Diagnostic and Prognostic Utility of Procalcitonin in Patients Presenting to the Emergency Department with Dyspnea (DPUP) study used the populations from the ProBNP Investigation of Dyspnea in the Emergency Department (PRIDE) study and the Biomonitoring and Cardiorenal Syndrome in Heart Failure (BIONICS-HF) study to assess the clinical utility of procalcitonin in diagnosing pneumonia in patients with dyspnea [9]. Blood samples from patients in these two studies were assessed for levels of procalcitonin, and the patients’ final diagnoses were reviewed. Of the 599 patients in the PRIDE study, only 365 had available samples for biomarker analysis, and of the 101 in the BIONICS-HF study, only 88 had available samples. From the possible 453 patients, a total of 60 had a diagnosis of pneumonia: 30 (6.5%) patients had a primary diagnosis of pneumonia and another 30 (6.5%) had a diagnosis of heart failure secondary to pneumonia.

The DPUP study found that procalcitonin results were increased in patients with either a primary or secondary diagnosis of pneumonia (0.38 vs. 0.06 ng/mL; P < .001). Also, the area under the receiver operating curve for the diagnosis of pneumonia using procalcitonin was 0.84 (95% confidence interval (CI), 0.77 - 0.91; P < .001). When a procalcitonin value of 0.10 ng/mL was used as the cutoff for diagnosis of pneumonia in patients with a previously uncertain cause of their dyspnea, the test was 80% sensitive and 77% specific. Also, when using the procalcitonin value in conjunction with other variables predictive of pneumonia, there was a reclassification improvement of 0.54 (95% CI, 0.24-0.82; P < .001) in diagnosing both true positive and true negative patients. 

The results of the DPUP study support the use of procalcitonin in the diagnosis of pneumonia in the emergency department (ED). However, the study only looked at patients in whom the ED clinician initially thought had a diagnosis of congestive heart failure. This limits the overall generalizability of the study because many patients who present to the ED with pneumonia will not present with a clinical picture similar to heart failure. The patients in this study may have had severe pneumonia or comorbid medical conditions that may have contributed to the value of this marker. However, this study is strongly supportive of the use of procalcitonin when trying to distinguish pneumonia from heart failure and may show this marker has great utility in patients who are difficult to distinguish between otherwise.

The Prediction of Blood Culture Results by Measuring Procalcitonin Levels and Other Inflammatory Biomarkers (PBC-PCI) study was a retrospective analysis of 422 samples collected at the life-saving emergency center of Tokyo Medical University Hachioji Medical Center in a 36 month time period [10]. The samples were only taken from patients with at least one of the following: Fever greater than 38^o^C, chills and shivering, and/or suspicion of bacterial or fungal infection. The biomarkers analyzed were procalcitonin level, white blood cell count (WBC), C-reactive protein (CRP) level, and platelet (PLT) count.

This paper will focus specifically on the results that directly relate to the diagnostic accuracy of procalcitonin in predicting blood cultures. The PBC-PCI study found that patients with a positive blood culture had significantly higher values of procalcitonin (30.62 ng/ L vs. 10.47 ng/ L; P < 0.001) than patients who had negative blood cultures. Also, when using a procalcitonin cutoff value of 0.5 ng/mL, a common level used for diagnosis of sepsis, patients above the cutoff showed significantly higher rates of positive blood cultures compared to patients with negative cultures (34% vs. 13.7%; P < .05, respectively). When using principal component analysis with the X axis (component 1) being bacterial (fungal) infection and the Y axis (component 2) being systemic inflammation, procalcitonin had the largest effect on component 1 compared to the other biomarkers analyzed. In contrast, procalcitonin showed the smallest impact on component 2 compared to the other factors.

Overall, the PBC-PCI study supports the use procalcitonin values, in conjunction with the other biomarkers, as an indication of bacterial infection. The usefulness of the procalcitonin, however, is still in question because, even though patients with values greater than 0.5 ng/mL had increased rates of positive blood cultures, the rate was only 34%, which is still rather small. Therefore, the value alone is not sufficient to indicate blood culture but, when increased, supports the use of empiric antibiotics until blood culture results return. In the context of sepsis, this study supports the notion that elevated procalcitonin is more indicative of bacterial infection rather than systemic inflammation.

A 2015 multinational, prospective, observational study in critical care by Schuetz, et al. assessed the use of procalcitonin and two other biomarkers as risk stratification in 7,132 ED patients across three different study centers in Switzerland, France, and the US. Procalcitonin was found to be strongly predictive of death within 30 days (area under the curve (AUC) 0.75), ICU admission (AUC 0.62), and high initial triage priority (AUC 0.58) [11]. These results were significant among several multivariate models grouped by age, gender, comorbidities, main symptom, main diagnosis, and vital signs.

The other two biomarkers included in this study were pro-adrenomedullin and copeptin, which were also found to be predictive of all three outcomes. Pro-adrenomedullin was found to be the most significant predictor (AUC 0.83, 0.67, and 0.67 for each outcome, respectively) [11]. Of the three biomarkers, procalcitonin is the one most specific for infection, whereas pro-adrenomedullin is classically a sign of inflammation and copeptin is one of stress. A possible reason for pro-adrenomedullin being the most predictive of death, ICU admission, and high initial triage priority is that inflammation is inclusive of a greater number of conditions than infection. Because patients with all different diagnoses were included and no infection-specific outcomes were measured, this study does not demonstrate procalcitonin as a way to diagnose bacterial infection but rather demonstrates its utility as a prognostic marker in an ED setting.

A 2016 retrospective study by Balk, et al. analyzed the differences in total hospital length of stay, ICU length of stay, and hospital costs of patients admitted to an ICU with either an admitting or discharge diagnosis of sepsis, septicemia, systemic inflammatory response syndrome (SIRS), or shock. Thirty-three thousand five hundred and ninety-two patients received procalcitonin testing within two days of ICU admission and 98,543 did not. Those receiving the procalcitonin testing had a decreased total length of stay (11.6 days vs 12.7 days; p < 0.001), ICU length of stay (5.1 days vs 5.3 days; p < 0.03), and hospital costs ($30,454 vs $33,213; p < 0.001) [12].

Because this analysis was limited to patients with a diagnosis along the spectrum of sepsis, it demonstrates procalcitonin’s utility in guiding treatment in the context of infection. The actual mortality outcome for patients receiving procalcitonin testing was higher (19% vs 18.3%), which may be due to illness severity and comorbidities that led providers to order procalcitonin testing in the first place. Because the indications for the procalcitonin testing were not noted in the database used in this study, we cannot definitively reconcile the increased mortality with the decrease in cost and length of stay, but limiting the analysis to survivors yields nearly identical results for the three outcomes [12].

Cost is a barrier to the widespread implementation of any diagnostic laboratory test, especially as the nation looks for ways to reduce the cost of healthcare. This study demonstrates procalcitonin testing as a way to decrease total hospital spending by almost $3,000, suggesting the money required to perform the test may be a worthy investment. This decreased hospital cost is very likely related to the shorter length of ICU and total stay and suggests that routine implementation of procalcitonin in patients with sepsis can guide clinical decision making in a way that reduces unnecessary tests, procedures, and admission length.

## Conclusions

Septicemia is a growing problem in the US that could be reduced by the use of procalcitonin markers. It could decrease sepsis, total medical costs, and guide antibiotic therapy. The DPUP study, PBC-PCI study, and study by Schuetz, et al., supported the use of procalcitonin when determining a bacterial infection. This can be in the setting of distinguishing between bacterial and non-bacterial in an ED setting, or whether the patient may have positive blood cultures and needs more management of their bacterial infection. Implementation of procalcitonin in patients can guide clinical decisions to diagnose bacterial infections early on to reduce unnecessary tests, procedures, and hospital stay length. This would overall reduce mortality and total medical costs. Most studies were limited in their sample of patients, which could affect the generalizability of the use of procalcitonin as a marker of infection. There is a need for further research to determine whether or not the routine use of procalcitonin should be used in settings other than the emergency department.
